# Assessing the value of phenotypic information from non-genotyped animals for QTL mapping of complex traits in real and simulated populations

**DOI:** 10.1186/s12863-016-0394-1

**Published:** 2016-06-21

**Authors:** Thaise P. Melo, Luciana Takada, Fernando Baldi, Henrique N. Oliveira, Marina M. Dias, Haroldo H. R. Neves, Flavio S. Schenkel, Lucia G. Albuquerque, Roberto Carvalheiro

**Affiliations:** UNESP, Universidade Estadual Paulista, Faculdade de Ciências Agrárias e Veterinárias, Jaboticabal, 14884-900 São Paulo, Brazil; GenSys Consultores Associados S/C Ltda, Porto Alegre, 90680-000 Brazil; Centre for Genetic Improvement of Livestock, University of Guelph, Guelph, N1G2W1 ON Canada

**Keywords:** GBLUP, GWAS, Single-step

## Abstract

**Background:**

QTL mapping through genome-wide association studies (GWAS) is challenging, especially in the case of low heritability complex traits and when few animals possess genotypic and phenotypic information. When most of the phenotypic information is from non-genotyped animals, GWAS can be performed using the weighted single-step GBLUP (WssGBLUP) method, which permits to combine all available information, even that of non-genotyped animals. However, it is not clear to what extent phenotypic information from non-genotyped animals increases the power of QTL detection, and whether factors such as the extent of linkage disequilibrium (LD) in the population and weighting SNPs in WssGBLUP affect the importance of using information from non-genotyped animals in GWAS. These questions were investigated in this study using real and simulated data.

**Results:**

Analysis of real data showed that the use of phenotypes of non-genotyped animals affected SNP effect estimates and, consequently, QTL mapping. Despite some coincidence, the most important genomic regions identified by the analyses, either using or ignoring phenotypes of non-genotyped animals, were not the same. The simulation results indicated that the inclusion of all available phenotypic information, even that of non-genotyped animals, tends to improve QTL detection for low heritability complex traits. For populations with low levels of LD, this trend of improvement was less pronounced. Stronger shrinkage on SNPs explaining lower variance was not necessarily associated with better QTL mapping.

**Conclusions:**

The use of phenotypic information from non-genotyped animals in GWAS may improve the ability to detect QTL for low heritability complex traits, especially in populations in which the level of LD is high.

## Background

Despite advances in genome-wide association study (GWAS) approaches, QTL detection using real data is still challenging, especially in unfavorable scenarios such as those posed by low heritability complex traits and by the availability of relatively few genotyped animals.

Bayesian multiple-regression models are the preferred methods for GWAS [1]. In addition to allowing the simultaneous inclusion of all markers in the analyses, Bayesian methods permit to consider different prior distributions for marker effects, an appealing quality especially for traits affected by large QTL. In a simulation study, Van den Berg et al. [2] tested the adequacy of Bayesian multiple-regression methods for QTL mapping and observed that Bayes C [3] is suitable to detect QTL, especially in the case of traits with medium to high heritability and a large number of records.

The single-step GBLUP method (ssGBLUP) [4, 5], originally used in plant and animal breeding for the prediction of breeding values, has also been applied to GWAS [6, 7]. Despite the benefits of ssGBLUP compared to multiple-step methods [8–10], it is based on an infinitesimal model, i.e., a model in which all markers are assumed to have a small effect. This model is particularly limiting when traits affected by large QTL are analyzed. Given this limitation, Wang et al. [6] proposed the weighted single-step GBLUP (WssGBLUP) method, which permits to combine all available pedigree, phenotypic and genotypic information in a single step, weighting the marker effects according to their (supposed) importance for the trait of interest. In WssGBLUP, the use of phenotypes is not restricted to those of genotyped animals or to pseudo-phenotypes (e.g., daughter yield deviation) as in multiple-step methods, which is believed to be advantageous [6, 7]. However, it is not clear to what extent the use of phenotypes from relatives without genotypes affects QTL detection.

The aims of the present study were: 1) to determine whether additional phenotypic information from non-genotyped animals affects QTL mapping of real data, and 2) to evaluate, by simulation, whether this additional phenotypic information enhances QTL mapping for a low heritability complex trait and whether factors such as the extent of linkage disequilibrium (LD) in the population and weighting SNPs in WssGBLUP affect the importance of using information from non-genotyped animals in GWAS.

## Methods

### Phenotypes and pedigree

The set of real data contained the phenotypic information of age at first calving (AFC) of Nelore heifers controlled by the DeltaGen® breeding program. This dataset was chosen to represent the scenario of a low heritability complex trait, with few available genotyped animals and a relatively large number of phenotypes.

The dataset contained records from animals raised on tropical pasture in different commercial herds located in the southeastern, western and central regions of Brazil. During the breeding season, usually the rainy period, the heifers are artificially inseminated or naturally mated. In general, the first mating of heifers occurs at about 26 months of age, although some herds expose heifers earlier at an age of about 14–18 months. Two scenarios were established to perform GWAS of AFC. The first scenario included all heifers that had the opportunity to breed at an early age and with observed AFC (SI), totaling 43,482 females, and the second considered only heifers with observed AFC and available genotype (SII), totaling 1,813 females. The pedigree file used in the two scenarios contained 237,602 animals; of these, 100,697 had known parents.

### Genotyped animals

A total of 1,829 Nelore females were genotyped using the Illumina Bovine HD panel (Illumina®, San Diego, CA, USA), which contains 777,962 single nucleotide polymorphism (SNP) markers. These females were born between 2007 and 2009 and were chosen among contemporary heifers exposed at an early age (14–18 months), which belonged to two main farms of the commercial breeding program. Heifers that did not conceive in the anticipated breeding season were also exposed in the regular breeding season, at about 26 months of age. Heifers that did not conceive in either season were excluded from the study. The minimum, maximum and average values of observed AFC of the genotyped females were 698, 1,200 and 1,050 days, respectively, and the proportion of heifers considered precocious (AFC < 900 days) was 28.2 %.

Quality control (QC) of the genotypes was performed discarding information of SNPs mapped in non-autosomal regions (42,669) and SNPs with a GC score < 0.7, a call rate < 0.95 (16,484), a minor allele frequency (MAF) < 0.02 (266,502), and a p-value of the Hardy-Weinberg equilibrium test < 10^−5^ (6,925). Fifty-four SNPs presenting duplicated map coordinates with other SNPs were also excluded from the dataset. To avoid redundancy of genotypic information, SNPs that were highly correlated (r^2^ > 0.995) with other SNPs within a sliding window containing 100 consecutive markers were removed from the dataset. Only one marker of each pair of highly correlated SNPs was discarded (111,450). Samples with a call rate < 0.9 for SNPs passing QC were excluded (16). The remaining numbers of SNPs and samples after QC were 333,878 and 1,813, respectively.

### Simulated data

The number of phenotypes and genotypes available in the simulation study mimics the information available in the real dataset. The phenotypes and genotypes were simulated using the QMSim v.1.10 software [11]. Ten replicates of a hypothetical trait with a heritability of 0.14 and phenotypic variance of 1.0 were performed. This heritability was chosen since it was the estimate obtained with the real data.

### Simulated population

Two different populations, which differed in the number of historical generations, were simulated to produce different levels of LD. In both cases, a historical population was generated from generation zero to 1,000, with a constant size of 1,000 animals. From generation 1,001, a gradual reduction in the number of animals (from 1,000 to 200) was simulated, producing a bottleneck effect and, consequently, genetic drift and LD. This gradual reduction occurred over 1,020 or 2,020 generations, resulting in a population with a lower (LLD) or higher (HLD) level of LD to mimic levels of LD in taurine [12] and indicine [13] cattle, respectively. The simulation process of LLD and HLD only differed in the number of historical generations. The 200 animals (100 females) of the last generation of the historical population were selected for the expanded population. The size of this population mimicked the effective size of the real population [14]. For the expanded population, a mating system based on the random union of gametes, absence of selection and an exponential growth of the number of females (the number of dams was doubled every generation) were considered, with a replacement rate of 100 % every generation and an average of five products per dam, with equal probability of being a male or a female. Thus, the 100 dams of the last generation of the historical population produced 500 offspring (first generation of the expanded population); of these, 200 females were selected to be the dams of the next generation of the expanded population and so forth. Six expanded generations were generated, resulting in 16,000 animals in the last generation of the expanded population (8,000 females). After the expansion process, 240 males and 6,000 females of the last generation were randomly selected to comprise the founder animals of the selection population. The selection population covered 15 generations. In each generation of the selection population, the selected males and females were randomly mated, generating a single product with equal probability of being a male or a female. The replacement rate of sires and dams was kept constant at 20 % and the selection criterion was the expected breeding value. A total of 90,000 animals were produced over the 15 generations, mimicking the number of available phenotypes in the real dataset; 50 % were females whose phenotypic data were used for GWAS. The genotypes of 2,000 females of the last three generations (13, 14 and 15) were randomly selected for GWAS.

### Simulated genome and phenotype

It was assumed that QTLs explain 100 % of genetic variance. The simulated genome had a total length of 2,333 cM, 735,293 markers, and 7,000 QTLs. The number of markers and QTLs per chromosome ranged from 46,495 to 12,931 and from 121 to 438, respectively, and were randomly distributed over 29 autosomes. All markers were bi-allelic, mimicking SNPs found in commercial bovine panels. For the QTLs, the number of alleles per loci ranged (randomly) from 2 to 4.

QTL allele effects were sampled from a gamma distribution with a shape parameter of 0.4 [15] and a scale parameter determined internally with QMSim, obeying the simulated genetic variance. A mutation rate of 10^−4^ for markers and QTLs in the historical population was considered. The number of mutations was sampled from a Poisson distribution with mean *u* (u = 2*number of loci*mutation rate) and each mutation was assigned to a random locus in the genome. A total of 335,000 markers (MAF ≥ 0.02) and 1,000 segregating QTLs were randomly selected from the last generation of the historical population to generate genotypic data for the selection population. The average distance between adjacent markers was 0.007 cM. Although the genetic architecture of reproductive traits is unknown, the simulation parameters used in this study aimed to mimic a polygenic complex trait affected by many genes of small effects and by few genes with more pronounced effects. The phenotypes of the animals comprised the sum of QTL effects plus an error term sampled from a normal distribution with zero mean and variance of 0.86, resulting in a trait with a heritability of 0.14.

### Linkage disequilibrium analysis

The LD between any two loci on the same chromosome was assessed by the r^2^ measure [16] using the SnppldHD software (Dr. Sargolzaei, University of Guelph, Canada). The pattern of LD decay of the real and simulated data was compared to evaluate the adequacy of the simulation process.

### Statistical analysis

Two statistical methods were used for real and simulated data, namely WssGBLUP [6] and Bayes C [3]. Although comparison of the methods was not part of our objective, Bayes C was also used since it has been suggested as a suitable method for QTL detection [2].

For real data, the WssGBLUP method adopted was based on the following model: *y* = *Xβ* + *Z*_*a*_ *a* + *e*, where y is the vector of phenotypic observations (AFC, in days); *X* is an incidence matrix relating phenotypes to fixed effects; *β* is the vector of fixed effects, including contemporary group (defined by the concatenation of classes for herd, year, season and weaning and yearling management groups, containing on average 80.52 heifers) and age of heifer’s dam as covariate (linear and quadratic effects); *Z*_*a*_ is an incidence matrix that relates animals to phenotypes; *a* is the vector of direct additive genetic effects, and *e* is the vector of residuals. The covariance between *a* and *e* was assumed to be zero and their variances were considered to be *Hσ*_*a*_^2^ and *Iσ*_*e*_^2^, respectively, where *σ*_*a*_^2^ and *σ*_*e*_^2^ are the direct additive and residual variance, respectively; *H* is the matrix which combines pedigree and genomic information [8], and *I* is an identity matrix. Solutions for this model were obtained by replacing the inverse of the regular relationship matrix (*A*^*−1*^) in the mixed model equations with the inverse of *H* (*H*^*−1*^) [4, 8]. The analyses were performed using the BLUPF90 family programs [17].

To assess the value of phenotypic information from non-genotyped animals in QTL mapping, the above model was applied considering all available phenotypes in scenario SI and only the phenotypes of genotyped cows in scenario SII. Since fixed effects would not be as well estimated in SII as in SI, to fairly compare the scenarios the phenotypic observations in SII were pre-adjusted for the fixed effect estimates obtained in a previous “regular” BLUP analysis considering all available phenotypes. Thus, vector *β* of the GWAS in SII contained only an overall mean and vector *y* the pre-adjusted AFC. The “regular” BLUP analysis used the same model as described above, except that the variance of *a* was assumed to be *Aσ*_*a*_^2^, where *A* is the regular numerator relationship matrix.

For the simulated data, the same WssGBLUP model was used, except that only an overall mean was considered as fixed effect. Since the contemporary group effect was not simulated, there was no need to pre-adjust the phenotypes in scenario SII for the simulated data.

For both scenarios (SI and SII) and datasets (real and simulated), the solutions of SNP effects (*û*) were obtained according to VanRaden et al. [18] and Stranden & Garrick [19]: *û* = *DP* ’ [*PDP* ’]^− 1^*â*_*g*_, where *D* is a diagonal matrix with weights for SNPs; *P* is a matrix relating the genotypes of each locus, and *â*_*g*_ is the vector of predicted breeding values of genotyped animals. Matrix *D*, direct additive genetic effects and SNP effects were iteratively recomputed according to the method referred to as “ssGBLUP/S2” by Wang et al. [6]. In the first iteration, the diagonal elements of *D* (*d*_*i*_) were assumed to be 1 (i.e., the same weight for all markers). For the subsequent iterations, *d*_*i*_ was calculated as: *d*_*i*_ = *û*_*i*_^2^p_i_(1- p_i_), where *û*_*i*_ is the allele substitution effect of the *i*^th^ marker estimated from the previous iteration, and *p*_i_ is the allele frequency of the second allele of the *i*^th^ marker. Before the beginning of a new iteration, matrix *D* was normalized to ensure that the total genetic variance was constant across iterations. Three iterations (w1, w2 and w3) were performed for each scenario, resulting in an increasing shrinkage from w1 to w3 for the SNPs explaining lower variance and, consequently, in an increasing proportion of variance being explained by the remaining markers. According to Wang et al. [7], three iterations are sufficient to reduce the noise of unimportant markers, i.e., to shrink their effects toward zero. Thus, the notation SIw1 refers to scenario SI using weight w1, SIw2 to scenario SI using w2, and so forth. An equivalent notation was adopted for scenario SII.

Bayes C analysis was based on the following model [3]: $y = 1\mu + {\displaystyle \sum_{i=1}^n}{g}_i{b}_i{\delta}_i + e$, where vectors *y* and *e* are the same as described above; *1* is a vector of ones; *μ* is the overall mean*; g*_*i*_ is the vector containing the genotypes of the animals for the *i*^th^ SNP; *b*_*i*_ is the allele substitution effect of the *i*^th^ SNP, and *δ*_*i*_ is an indicator variable (0, 1) sampled from a binomial distribution with parameters *n* and *π*, where *n* is the number of SNPs and *π* is the fraction of SNPs not included in the model. For the real dataset, a prior beta distribution with parameters *α* = 10^8^ and *β* = 10^10^ was assumed for *π* so that, in practice, *π* was almost fixed to 0.99 [20]. For the simulated datasets, *π* assumed two possible values, almost fixed to 0.99 as used for the real data, or almost fixed to 0.999 assuming a prior beta distribution with parameters *α* = 10^8^ and *β* = 10^11^. This strategy, rather than letting *π* be estimated from the data (Bayes C*π*), was adopted because it was found to provide better results in QTL detection [2]. A scaled inverse chi-squared prior distribution was assumed for the variance of SNP effects (*σ*^*2*^_*g*_) and for the residual variance (*σ*^*2*^_*e*_).

The SNP genotypes were coded as the number of copies of one of the SNP alleles, i.e., 0, 1, or 2. For Bayes C, only scenario SII was evaluated because the adopted implementation did not allow the inclusion of phenotypes from non-genotyped animals. As in scenario SII of WssGBLUP, vector *y* in Bayes C contained the pre-adjusted phenotypes of AFC. Bayes C analysis was performed using the Markov chain Monte Carlo algorithm implemented in the GS3 software [20], running a single chain with 550,000 iterations, a burn-in period of 50,000, and a thinning interval of 50 iterations.

### Criteria for comparison

For the real data, the GWAS results were compared based on SNP effect estimates and on the proportions of variance explained by SNPs within consecutive 1-Mb windows. A total of 2,525 windows spanning all autosomes were considered, with an average density of 132 ± 44 SNPs per window. The top 10 windows that captured the highest proportion of variance explained by the markers were identified for the different scenarios and methods. The QTLdb database [21] was consulted to assess the existence of previously described QTL related to reproductive traits and overlapping the top 10 windows and their neighboring 1-Mb windows (next to the left and to the right). The UMD3.1 bovine genome assembly [22] was used as the reference map. The presence of a previously described QTL in the QTLdb database was double checked in the originally listed references. Recent scientific publications were also manually searched with the same purpose. The SNPchiMp database [23] was used to standardize the reference assembly (UMD3.1) across studies.

For the simulated data, the following statistics were calculated: number of QTLs explaining 1 % or more of the genetic variance (topQTL); number of top 1-Mb marker windows accounting for the highest proportion of variance explained by the markers (topMRKw) - this number was set equal to topQTL so that the different methods could be compared on the same basis; sum of the percentages of genetic variances explained by all topQTL (Pvar_topQTL) and top marker windows (Pvar_topMRKw); highest percentage of genetic variance explained by a topQTL (Pvar_1^st^QTL) and a top marker window (Pvar_1^st^MRKw), and the estimated number of true QTLs (NtrueQTL), i.e., the number of topQTL identified by a topMRKw located no more than 1 Mb from a true QTL position.

## Results and discussion

### Linkage disequilibrium

**Fig. 1:**
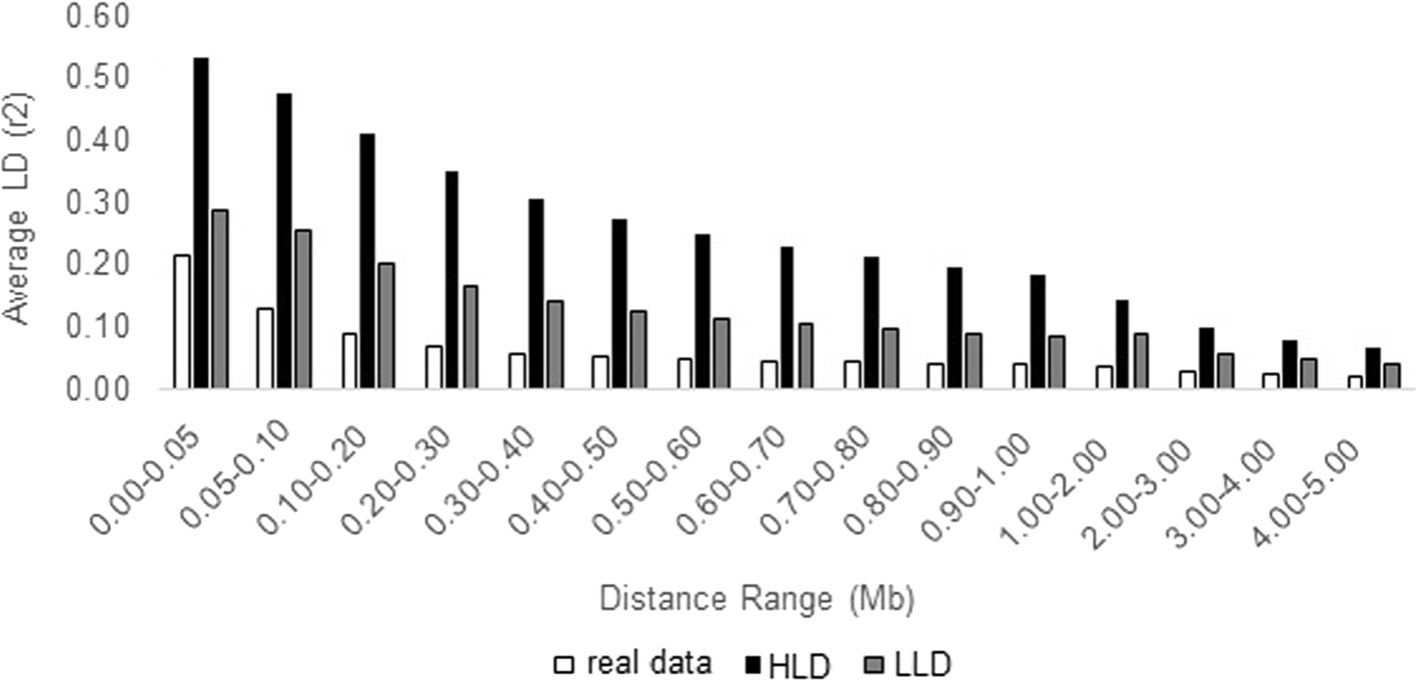
Linkage disequilibrium (LD) decay of real data and two simulated populations (HLD and LLD). Average LD, expressed in r^2^, according to varying distances between markers (Mb)

Figure 1 shows the average LD decay for the real and simulated HLD and LLD populations. The LD of the simulated population with HLD was closer to the pattern presented by taurine breeds [12] and was a consequence of the large number of historical generations (1,001 to 3,020) simulated to produce the bottleneck effect. For the simulated LLD population, the LD was slightly higher than that obtained with the real data. However, it was similar to the levels of LD observed by Espigolan et al. [13] in another set of Nelore animals, indicating adequacy of the LLD population to represent real indicine populations.

### Simulated data

The simulation process resulted in an average number of QTLs explaining 1 % or more of the genetic variance (topQTL) of 16.7 in the HLD population (Table 1). Taken together, the topQTL explained 29.74 % of the genetic variance, with the most important QTL explaining on average 5.07 %. As shown in Table 1, the top marker windows captured a smaller proportion of the genetic variance when compared to the topQTL, except for the analyses considering stronger shrinkage on SNPs explaining lower variance (w3) or a lower proportion of SNPs included in the model (π = 0.999). These results are related in part to the imperfect LD between markers and QTLs and to false positive signals being captured by the markers.

**Table 1:** Average (SD) of marker and QTL related statistics of the simulated population presenting high LD

Method/Scenario^a^	Pvar_topMRKw(%)^b^	Pvar_1^st^MRKw(%)^c^	NtrueQTL^d^
Bayes C (π = 0.99)	7.78 (0.99)	1.19 (0.63)	2.20 (1.23)
Bayes C (π = 0.999)	46.13 (14.13)	16.45 (17.86)	1.90 (1.29)
WssGBLUP/SIw1	5.16 (0.74)	0.54 (0.17)	2.90 (1.66)
WssGBLUP/SIw2	17.03 (3.11)	2.29 (0.94)	2.90 (1.79)
WssGBLUP/SIw3	38.07 (6.27)	7.30 (3.30)	1.30 (1.16)
WssGBLUP/SIIw1	5.30 (0.63)	0.59 (0.19)	2.00 (1.49)
WssGBLUP/SIIw2	18.76 (1.70)	2.65 (0.93)	1.90 (1.29)
WssGBLUP/SIIw3	39.31 (7.47)	7.82 (5.80)	0.90 (0.74)
True values	Pvar_topQTL(%)^b^	Pvar_1^st^QTL (%)^c^	NtopQTL^d^
	29.74 (4.88)	5.07 (2.36)	16.7 (2.83)

The averages are expressed over the ten replicates, using the Bayes C and weighted single step GBLUP (WssGBLUP) analyses

^a^GWAS using (SI) or ignoring (SII) phenotypic information of non-genotyped animals, applying different weights (w1, w2 and w3) for the SNP effects in the WssGBLUP method. And using π = 0.99 and π = 0.999 in the Bayes C method

^b^Genetic variance (%) explained by the sum of variances accounted by top marker windows (Pvar_topMRKw) and by the NtopQTLs (Pvar_topQTL)

^c^Maximum genetic variance (%) explained by a top marker window (Pvar_1^st^MRKw) and by a topQTL (Pvar_1^st^QTL)

^d^Estimated number of true QTLs explaining 1 % or more of the genetic variance (NtopQTL), and number of NtopQTLs identified by a top marker window distant no more than 1 Mb from a NtopQTL (NtrueQTL)

As expected, stronger shrinkage on SNPs explaining lower variance (from w1 to w3) and a higher proportion of SNPs assumed unimportant *a priori* (π = 0.99 to π = 0.999) resulted in higher variance that could be explained by the top marker windows. The method providing the highest proportion of variance explained by the top marker windows was Bayes C (π = 0.999) (Table 1).

In general, irrespective of the method or scenario, GWAS exhibited poor ability to map the topQTL (maximum of 2.9 out of 16.7 true QTLs; Table 1), indicating that the available phenotypic and genotypic information of the simulated population was not sufficient to properly map the QTLs. These results are in agreement with Van den Berg et al. [2] who, in a simulation study, also observed poor identification of QTLs for traits with low heritability, a large number of QTLs and few records.

As can be seen in Table 1, the analyses using different weights (w1 and w2) exhibited a similar ability to map the QTLs. Furthermore, a higher percentage of variance being explained by the top marker windows as a result of stronger shrinkage on SNPs explaining lower variance was not necessarily associated with better performance of QTL mapping. For the HLD population, the WssGBLUP analyses using w1 and w2 tended to outperform the analysis using w3 in terms of the detection of true QTLs. Using a higher π also did not improve the ability of Bayes C to detect QTLs (Table 1). It is important to emphasize that the ability of different methods to map QTL depends on the genetic architecture of the trait; thus, our results cannot be extrapolated, for example, to traits suspected to be affected by major gene(s).

The use of phenotypic information from non-genotyped animals tended to increase the precision of QTL detection, especially in the HLD population. The analysis with the best result considering additional phenotypic information was able to detect on average 17.4 % (2.9 out of 16.7) of the topQTL, whereas WssGBLUP analysis ignoring this information detected a maximum of 12.0 % (2.0 out of 16.7) of the topQTL (Table 1). A possible explanation for this result is that phenotypic information from non-genotyped animals helps predict the genetic merit of genotyped animals (*â*_*g*_) more precisely through relatedness. Since the SNP effects estimated with the WssGBLUP method are computed as a function of *â*_*g*_, better predictions of *â*_*g*_ would ultimately result in better estimation of SNP effects.

For the LLD population, the importance of using additional phenotypic information became less evident. As shown in Table 2, although slightly better on average, the ability of the SI scenario to detect QTLs (NtrueQTL) was similar to that of the SII scenario. This finding might be explained by the fact that animals of the LLD population are to some extent less related than animals of the HLD population. Thus, phenotypic information from non-genotyped animals does not contribute to improve the prediction of genetic merit of genotyped animals and, consequently, QTL mapping.

**Table 2:** Average (SD) of marker and QTL related statistics of the simulated population presenting low LD

Method/Scenario^a^	Pvar_topMRKw(%)^b^	Pvar_1^st^MRKw(%)^c^	NtrueQTL^d^
Bayes C (π = 0.99)	3.95 (0.58)	0.42 (0.10)	1.40 (0.97)
Bayes C (π = 0.999)	36.71 (12.35)	12.39 (11.93)	1.80 (1.62)
WssGBLUP/SIw1	2.73 (0.53)	0.25 (0.08)	2.00 (1.70)
WssGBLUP/SIw2	10.58 (1.59)	1.45 (0.37)	2.10 (1.29)
WssGBLUP/SIw3	26.80 (4.93)	4.51 (1.31)	1.20 (0.63)
WssGBLUP/SIIw1	2.82 (0.35)	0.26 (0.04)	1.90 (1.20)
WssGBLUP/SIIw2	12.05 (1.72)	1.69 (0.38)	1.90 (1.10)
WssGBLUP/SIIw3	31.60 (3.76)	5.66 (2.49)	1.10 (0.88)
True values	Pvar_topQTL(%)^b^	Pvar_1^st^QTL (%)^c^	topQTL^d^
	24.32 (4.92)	3.19 (0.61)	15.4 (2.32)

The averages are expressed over the ten replicates, using the Bayes C and weighted single step GBLUP (WssGBLUP) analyses

^a^GWAS using (SI) or ignoring (SII) phenotypic information of non-genotyped animals, applying different weights (w1, w2 and w3) for the SNP effects in the WssGBLUP method. And using π = 0.99 and π = 0.999 in the Bayes C method

^b^Genetic variance (%) explained by the sum of variances accounted by top marker windows (Pvar_topMRKw) and by the NtopQTLs (Pvar_topQTL)

^c^Maximum genetic variance (%) explained by a top marker window (Pvar_1^st^MRKw) and by a topQTL (Pvar_1^st^QTL)

^d^Estimated number of true QTLs explaining 1 % or more of the genetic variance (NtopQTL), and number of NtopQTLs identified by a top marker window distant no more than 1 Mb from a NtopQTL (NtrueQTL)

The number of QTLs explaining 1 % or more of the genetic variance (topQTL) in the LLD population was 15.4. Taken together, the topQTL explained 24.32 % of the genetic variance, with the most important QTL explaining on average 3.19 %. Compared to the HLD population, all methods and scenarios involving the LLD population exhibited a poorer ability to detect true QTLs. In addition, the pattern of the Bayes C results obtained for the LLD population differed from that of the HLD population. The average NtrueQTL of the analyses assuming π = 0.999 was slightly higher than the value obtained with the analyses using π = 0.99 (Table 2). These results reinforce that the adequacy of a method to detect QTL depends on the level of LD in the population of interest.

### Real data

**Fig. 2:**
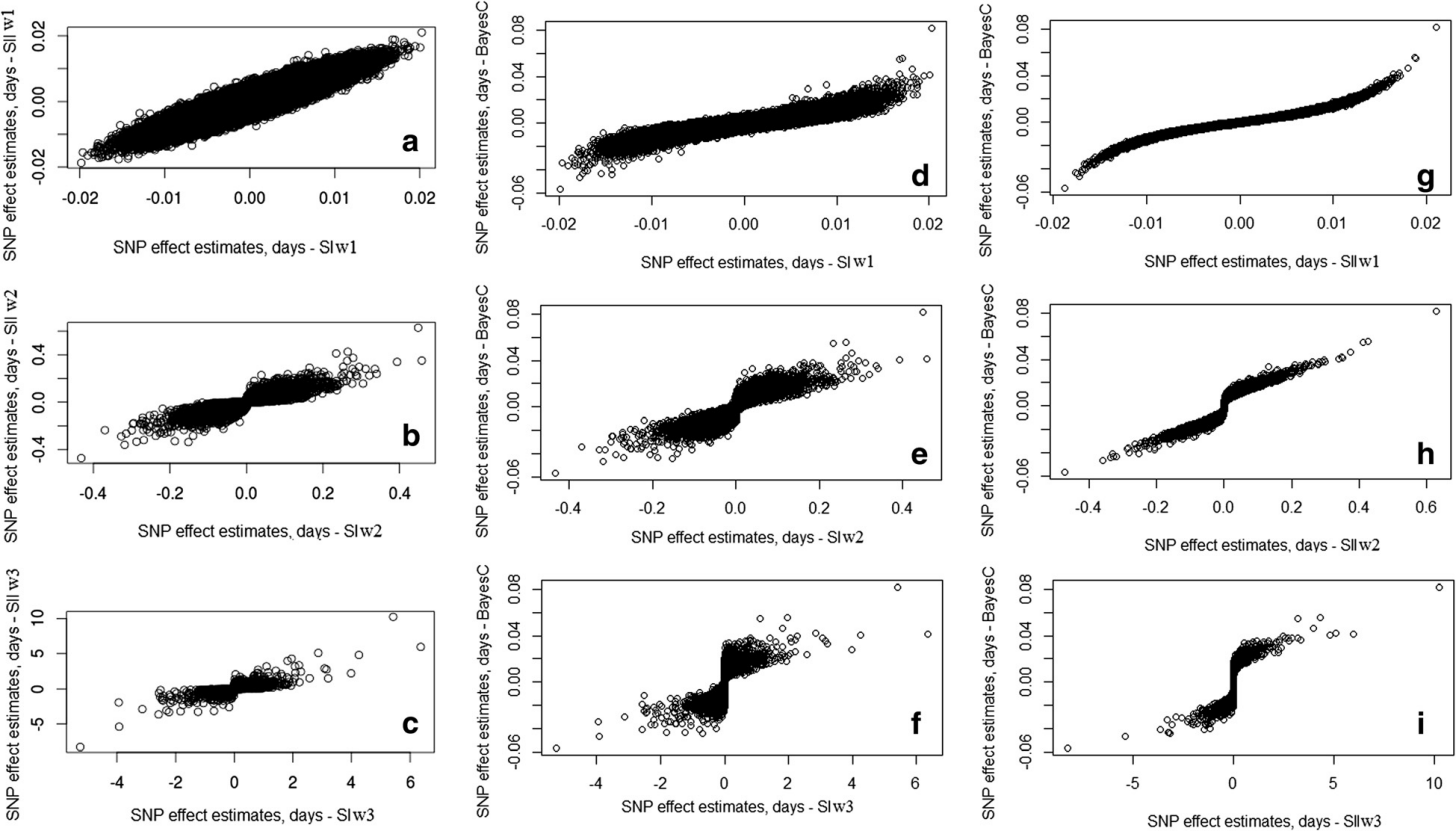
Scatter plots of SNP effect estimates for GWAS of age at first calving in Nelore cattle. The plots are for Bayes C and Weighted single-step GBLUP methods, considering (SI) or ignoring (SII) phenotypes from non-genotyped animals, under different weights (w1, w2 and w3) for the SNPs. **a** = SIw1 x SIIw1; **b** = SIw2 x SIIw2; **c** = SIw3 x SIIw3; **d** = SIw1 x BayesC; **e** = SIw2 x BayesC; **f** = SIw3 x BayesC; **g** = SIIw1 x BayesC; **h** = SIIw2 x BayesC; **i** = SIIw3 x BayesC

As observed for the simulated data, the addition of phenotypic information of non-genotyped animals also influenced the estimates of SNP effects for the real data (Fig. 2a, b and c). Although correlated, the SNP effects estimated with WssGBLUP were not the same for scenarios SI and SII. The influence of using or ignoring the phenotypes of non-genotyped animals became stronger as the shrinkage on SNPs explaining lower variance increased. For SNPs with more pronounced effects, the SNP effects estimated with WssGBLUP in SI were more similar to those of SII in w1 (Fig. 2a) compared to w2 (Fig. 2b) and w3 (Fig. 2c). This occurred because within each scenario the weights of further iterations were calculated as a function of the SNP effects estimated in the previous iteration [6]. Consequently, the differences became greater in each subsequent iteration.

As observed by Wang et al. [6] and in our simulation study, the total genetic variance was distributed for a smaller number of SNPs as the SNP effects were recomputed in the WssGBLUP method. Consequently, solutions from w1 (Fig. 2a, d, and g) were similar to those expected for a trait following an infinitesimal model and solutions from w3 (Fig. 2c, f, and i) were similar to those expected for an oligogenic trait. Unfortunately, when analyzing real data, the WssGBLUP method does not allow to infer which weight resulted in better estimates. Wang et al. [6] recognized that their method calculates the weights in a suboptimal manner and suggested some refinements, for example, the use of Bayesian methods.

According to Gianola et al. [24], unraveling the genetic architecture underlying a trait is not trivial even when Bayesian methods are adopted, since the priors dominate the inference in the case of few observations and when a large number of SNP effects need to be estimated simultaneously. This statement is supported by the simulation results of our study in which the prior value assumed for π determined the shrinkage on SNP effect estimates. Estimation of π from the data does not seem to be a good strategy for GWAS purposes [2].

The Bayes C solutions were more similar to the WssGBLUP solutions of scenario SII (Fig. 2g to i), in which the same phenotypic information was used, than those of scenario SI (Fig. 2d to f). For example, the association between Bayes C and WssGBLUP SIIw1 solutions was very high (Fig. 2g). This result suggests that, for the present study, the use or not of additional phenotypic information had a greater influence on SNP effect estimates than on the difference in the method. This evidence was not as strong for the simulated data, particularly for the LLD population. The sigmoidal shape of the graphs in Fig. 2e, f, h and i indicates that weights w2 and w3 applied in the WssGBLUP method resulted in more SNPs with their effects shrunk to zero than Bayes C using π ≈ 0.99.

As also observed in the simulation study, stronger shrinkage (w2 and w3) on SNPs explaining lower variance redistributed the variance and resulted in larger variances that are explained by the (supposedly) most important regions (Table 3). In SI, the proportion of variance explained by the top 10 windows was 1.83 %, 7.18 % and 23.65 % for w1, w2 and w3, respectively. These proportions were 1.84 %, 8.07 % and 34.70 % in SII. For the Bayes C method, the top 10 windows explained 2.65 % of the genetic variance.

**Table 3:** Top 10 windows explaining the highest proportion of variance of age at first calving

Rank	Bayes C	w1		w2		w3	
		SI	SII	SI	SII	SI	SII
	Ch/Wi/pvar^1^	Ch/Wi/pvar	Ch/Wi/pvar	Ch/Wi/pvar	Ch/Wi/pvar	Ch/Wi/pvar	Ch/Wi/pvar
1^st^	**18/5/0.40**	**18/5/0.23**	**18/5/0.23**	**18/5/1.13**	**18/5/1.17**	13/71/4.59	**23/27/9.66**
2^nd^	**8/107/0.34**	5/16/0.19	**17/50/0.21**	**8/107/0.84**	**5/115/1.16**	**23/27/3.26**	12/3/6.43
3^rd^	**17/50/0.29**	**8/107/0.19**	23/25/0.20	6/108/0.80	**8/107/1.08**	12/3/3.24	13/71/3.42
4^th^	**5/115/0.28**	**7/4/0.19**	**8/107/0.20**	2/18/0.71	**23/27/0.94**	2/18/2.94	**5/115/3.14**
5^th^	**7/4/0.27**	**17/50/0.18**	**7/4/0.18**	22/2/0.68^a^	**7/4/0.78**	7/26/1.79	3/12/2.72
6^th^	3/28/0.24	23/28/0.17	23/29/0.18	**7/4/0.68**	**17/50/0.67**	4/77/1.77^a^	7/41/2.48
7^th^	**23/27/0.22**	5/13/0.17	3/28/0.16	24/09/0.68	6/44/0.61	3/12/1.75	2/18/2.39
8^th^	23/25/0.21	5/17/0.17	1/27/0.16^a^	**5/115/0.56**	14/63/0.59^a^	**5/115/1.50**	**7/4/1.60**
9^th^	14/63/0.20^a^	10/92/0.17^a^	23/28/0.16	6/16/0.56	12/3/0.55	6/108/1.45	21/23/1.57
10^th^	23/29/0.20	23/29/0.17	5/13/0.15	25/33/0.55	3/28/0.53	11/9/1.37	1/27/1.29^a^

The GWAS applied Bayes C method and weighted single step GBLUP considering (SI) or ignoring (SII) phenotypes from non-genotyped animals, under different weights (w1, w2 and w3) for the SNPs. ^1^Ch = chromosome; Wi = 1 Mb window within the chromosome; pvar = proportion of variance explained by the SNPs within the window. The most common windows (ranked as top 10 in at list four analysis) are highlighted in bold.

^a^Window (or neighboring window) with a previous described QTL for bovine sexual precocity direct and indirect traits

Although the most important genomic regions identified by the different analyses were not the same, some coincidence was observed. Considering all seven analyses, 31 different windows were indicated as top 10 (Table 3). The most common windows identified by the analyses were window 4-Mb on chromosome 7 (7/4) identified as top 10 in six of the seven analyses; 5/115, 8/107 and 18/5 identified as top 10 in five analyses, and 17/50 and 23/27 identified as top 10 in four analyses. WssGBLUP/SIIw2 and Bayes C were the only methods that identified the six most common regions within the top 10 windows.

Although the simulation results indicated a poor ability to detect QTLs, some regions identified as important in the present study using real data coincided with regions reported by different authors [25–28] for direct and indirect traits of bovine sexual precocity (Table 4). The identification of the same regions in different studies provides evidence that these regions are important for the trait of interest.

**Table 4:** Common regions reported by different authors for bovine sexual precocity direct and indirect traits

Ch/wi	Traits reported in the region/Breed	Authors
1/27	Age at first calving/Hanwoo	Hyeong et al. [25]
14/63	Age at first calving/Hanwoo	Hyeong et al. [25]
10/92	Heifer pregnancy/Angus	Peters et al. [26]
22/2	Non-return of daughters at 56 days after insemination/Holstein	Schrooten et al. [27]
4/77	Body condition score^a^/Brahman and crossbreed	Porto-Neto et al. [28]

Ch = Chromossome and wi = Window detected by Bayes C (π = 0.99) and wSSGBLUP (w1, w2 and w3) methods

^a^Body condition score was listed for indirectly affecting reproductive performance [30]

Other studies have also identified important genes. Waters et al. [29], studying the transcriptional regulation process in the uterine endometrium of beef heifers receiving special dietary supplementation, detected many genes that were clustered in similar functional groups. One of these genes is the TSPO gene, which was found in a cluster described as “potentially co-regulated genes with reproductive function and involved in steroid biosynthesis”. Considering the importance of steroids for the regulation of female reproduction, this gene could be an important candidate gene associated with AFC in Nelore cattle. The TSPO gene is located within window 115 of BTA5 (UMD3.1), which was identified as a top window in all analyses, except for those using w1 (Table 3).

## Conclusions

Additional phenotypic information from non-genotyped animals influences the results of GWAS. Despite some coincidence, the most important genomic regions for AFC in Nelore cattle, identified by analysis using or ignoring phenotypes from non-genotyped animals, were not the same. The simulation results indicated that the inclusion of all available phenotypic information, even that of non-genotyped animals, provides small improvement in the detection of QTLs in GWAS of low heritability complex traits. This improvement is expected to be less pronounced in populations in which the level of LD is low.
